# A comparative review of cell culture systems for the study of microglial biology in Alzheimer’s disease

**DOI:** 10.1186/1742-2094-9-115

**Published:** 2012-05-31

**Authors:** Branden Stansley, Jan Post, Kenneth Hensley

**Affiliations:** 1 Department of Neurosciences, University of Toledo Medical Center, Toledo, OH, USA; 2 Oklahoma School for Science and Mathematics, Oklahoma City, OK, USA; 3 Department of Pathology, University of Toledo Medical Center, Toledo, OH, USA; 4 MS 1090, University of Toledo Health Science Center, 3000 Arlington Avenue, Toledo, OH, 43614, USA

**Keywords:** Alzheimer’s disease, Microglia, Cell culture, Nitric oxide, Cytokines

## Abstract

Over the past two decades, it has become increasingly apparent that Alzheimer’s disease neuropathology is characterized by activated microglia (brain resident macrophages) as well as the classic features of amyloid plaques and neurofibrillary tangles. The intricacy of microglial biology has also become apparent, leading to a heightened research interest in this particular cell type. Over the years a number of different microglial cell culturing techniques have been developed to study either primary mammalian microglia, or immortalized cell lines. Each microglial system has advantages and disadvantages and should be selected for its appropriateness in a particular research context. This review summarizes several of the most common microglial cell culture systems currently being employed in Alzheimer’s research including primary microglia; BV2 and N9 retroviral immortalized microglia; human immortalized microglia (HMO6); and spontaneously immortalized rodent microglial lines (EOC lines and HAPI cells). Particularities of cell culture requirements and characteristics of microglial behavior, especially in response to applied inflammogen stimuli, are compared and discussed across these cell types.

## Introduction

Microglia are the resident phagocytes and innate immune cells of the brain. Over the past few decades, there has been an increased interest in microglia, as many investigators have recognized the importance of this cell in the homeostasis, as well as various pathologies, of the central nervous system (CNS). It is now widely accepted that clustered populations of reactive microglia are hallmarks of Alzheimer’s disease (AD), and these brain cells are likely to contribute to the mechanisms of neuronal damage and cognitive loss [1]. Furthermore, activated microglia have been associated with a variety of neurodegenerative diseases including AD, Parkinson’s disease (PD) and amyotrophic lateral sclerosis (ALS) [1-3].

Since first described by Pio del Rio-Hortega in 1932 [4], microglia origins and functions in the CNS have been extensively debated. The common consensus is that microglia cells are of hematopoietic origin [5], however the specific cell lineage remains uncertain. The colonization of CNS by microglia has been shown to take place during embryonic development in rodents, at a prenatal stage (E10 to E19) [6]. These findings support the idea that there is heterogeneity of the microglial cell origins in the CNS, with one population from myeloid/mesenchymal origin (not necessarily derived from monocytes) and a second population representing a developmental and transitory form of fetal macrophage (possibly monocyte), which is an amoeboid microglial cell as described in the postnatal brain of rodents [7]. Regardless of these cells origins, it is clear that they are actively sensing and contributing to the changing microcosm of the brain.

Microglia have activation states similar to that of macrophages and exhibit functional plasticity during activation states. The resting state, or ‘ramified’ state, is relatively inactive or ‘quiescent’ but seems to perform surveillance functions [8]. In addition to the resting state, there are two functionally distinct activation states, M1 and M2. The former is classically activated, for instance in response to IFN-γ or lipopolysaccharide (LPS), and produces neuronal injury by secreting the pro-inflammatory cytokines such as TNF-, IL-1β, and reactive oxygen species/reactive nitrogen species (ROS/NOS). In contrast, the amoeboid M2 state acts as an anti-inflammatory by blocking the release of pro-inflammatory cytokines, ingesting debris, promoting tissue repair and releasing neurotrophic factors [9]. By using *in vitro* culture techniques, researchers can find ways to simulate many homeostatic or pathological conditions by manipulating these states.

Microglia cultures have been described as early as the 1930s [10]; however, the use of cultures to study microglia function did not occur until after a method for obtaining and culturing large amounts of microglia was developed and improved upon. The increased yield and homogeneity of cells in culture allows for increased data output in a shorter time compared to most *in vivo* experiments. Also, these *in vitro* cultures present a beneficial tool to study the activation state, releasable factors, motility, and other crucial components that characterize microglia, which cannot be sufficiently examined *in vivo*.

Presently there are many models of microglia and microglia-like cell lines used to examine neuro-inflammatory phenomena. These include primary microglia cultures, and immortalized microglia cell cultures, which are either retrovirus transformed or non-retrovirus transformed. These culture models share similarities but are also separated by crucial differences that must be weighed when choosing an appropriate model for neurodegenerative research. The focus of this review is to compare cell models with regard to origins, methods of culturing specific cell lines, individual cell properties and differences, and technical strengths and weaknesses. Furthermore, given the prevalence of AD research involving amyloid-β (β), the ability of each microglia type to react to this molecule will be addressed.

### Primary microglia cultures

Primary microglia cultures are very prevalent in neuro-inflammatory research due to the similarities in phenotype to *in vivo* cells. These cells are most often derived from the cortex of a rat or mouse before or early after birth. In a process described by Giulian and Baker [8], amoeboid microglia cells can be isolated from the mammalian brain and grown in culture. They show that by using a specific process of adhesion and agitation of the cultured glial cells, a purified culture of approximately 95% enriched microglia can be obtained [8] (Table 1). This technique has been used extensively since its creation and, although sometimes slightly manipulated, remains a popular method for cultivating *in vitro* microglia. The advantages of using amoeboid microglia directly from an animal are the functional characteristics that these cells are endowed with, such as secretory products and cell surface markers, which closely resemble endogenous cells.

**Table 1:** Amoeboid microglia isolation procedure

**Step**	**Manipulation**	**Time**
1.	Dissection and mechanical dissociation of cerebral tissue from newborn rat	1 hour
2.	Incubate in flask with defined medium, 37 °C	1 day
3.	Agitation by rotary shaker, 180 rpm, 37 °C	15 hours
4.	Adhesion incubation in flask, 37 °C	1 to 3 hours
5.	Agitation by hand, 20 °C	2 to5 minutes
6.	Adhesion incubation in flask, 37 °C	1 to 3 hours
7.	Agitation by hand, 20 °C	2 to 5 minutes

The table is adapted from *Guilian and Baker,* 1986 [8]. The table describes a common procedure for obtaining primary microglial cells.

When characterized, these cells were observed to be homogeneously negative for glial fibrillary acidic protein (GFAP, an astrocyte marker); galactocerebroside (GC an oligodendrocyte marker); peroxidase activity (neutrophils); and positive for non-specific esterases, all of which are indicative of microglia cells [8]. When stimulated with LPS and cytokines, primary microglia cultures have been shown to release nitric oxide (·NO) via upregulation of inducible nitric oxide synthase (iNOS, NOS-II) and superoxide (O_2_·) anions via activation of NADPH oxidase (NOX) complexes [11]. Furthermore, primary microglia secrete a host of other anti- and pro-inflammatory factors when induced by stimulating agents. Reactive oxygen species (ROS) and reactive nitrogen species (RNS) are released upon activation, similar to monocytes, macrophages and neutrophils [12].

Specifically relating to AD, microglial research has found that the β oligomer, a known neurotoxin, may produce more damage to neurons indirectly by activating microglia [13]. Using *in vitro* cultures, subneurotoxic concentrations of Aβ oligomer (5 to 50 nM) caused an activation of primary microglia, stimulating proliferation and ·NO production [13]. Furthermore, it has been shown that primary microglia have the ability to phagocytose Aβ as a reaction against Aβ accumulation [14]. This is an example of the M2 activation state, which was discussed earlier. It has also been shown that Aβ phagocytosis by primary microglia can be inhibited by cytokines such as IL-1β, TNF-, and IFN-γ, which most likely shifts the cells into the pro-inflammatory M1 state [15].

The state of amoeboid microglia can change *in vitro* to a ramified state (more quiescent), whereby the cells exhibit elongation of processes, functional loss of ability to phagocytose, and decreased proliferation. This transition is accelerated by incubation with retinoic acid, an agent known to increase cellular differentiation [8]. This change in state is similar to the two different states seen *in vivo* and provides yet another dynamic when studying neuro-inflammatory processes *in vitro*.

The ability to measure these proteins and cell markers in this cell model is beneficial; however, the extensive preparations needed for each experiment makes this model more time consuming and perhaps less attractive as compared to other microglia lines that have shorter prep-time, but maintain similar cell properties. One such way of obtaining cells that have faster proliferation rates, have a more homogeneous population, and at a lower cost, is by genetically immortalizing the primary microglia.

### Retroviral immortalized microglia: BV2 and N9

With low cell number and the time consuming techniques needed to cultivate primary microglia cultures, options were investigated to yield a large number of cells quickly. Such immortalized cell lines can be generated by infecting the cells with a retrovirus. Two commonly used cell lines of this type are the BV2 and N9 microglia cell lines which are derived from rat and mouse, respectively. Both cell lines have been used extensively in research related to neurodegenerative disorders.

After successfully immortalizing murine macrophages from bone marrow via the *v-raf/v-myc* carrying retrovirus (J2) [16], Blasi’s research group adapted this same technique to form the BV2 microglia cells [17]. To develop this line, microglia were first purified by adhesion/agitation as described previously [8], then incubated overnight with control or J2 virus containing supernatants in cell specific complete medium. After three to four weeks of incubation, proliferating cells were observed in infected cultures, where non-infected cultures lost adherence and died [17].

The BV2 cells were assessed for microglia cell markers and tested 90% positive for nonspecific esterase activity and all lacked peroxidase activity. Furthermore, BV2 cells were positive for MAC-1 and MAC-2, but negative for MAC-3 antigens. Also, similar to primary microglia, they were negative for GFAP and GC antigens, markers for astrocytes and oligodendrocytes [18], respectively. Secretion of cytokines was assessed and it was found that when stimulated with LPS, levels of IL-1 increased dose dependently [17]. Also, given that these cells express phagocytic capabilities, it was demonstrated that Aβ(1–42) fibrils can stimulate phagocytosis by microglia in a time- and dose-dependent manner [19].

Even with the similarity to primary microglia, the cells do contain oncogenes that render them in some ways different from primary microglia, such as increased proliferation and adhesion, and increased variance of morphologies [20]. The validity of BV2 cells as a sufficient substitute for primary microglia has been debated, and as a result, a few studies comparing different microglia lines emerged. One such study was conducted by Horvath *et al.*[20], who compared primary rat microglia to the BV2 line with regard to activation markers, motility and releasable factors, such as ·NO and cytokines. It was observed that the primary microglia and BV2 cells both express Iba-1, a microglia activation marker. Furthermore, upon LPS stimulation, BV2 cells secreted lesser but still substantial amounts of ·NO compared to primary microglia [20].

The major idea that BV2 immortalized cells have similar functions as primary microglia, but not to the same extent, was further examined by Henn *et al.*[21]. This group examined the BV2 cells as an appropriate alternative to the primary cultures. They found that in response to LPS, 90% of genes induced by the BV2 cells were also induced by primary microglia; however, the up-regulation of genes in the BV2 was far less pronounced than in primary microglia [21].

With the BV2 cell line, there appears to be a trade-off of magnitude of stimulatory response, with preparation time and technical feasibility between experiments. Another popular retroviral-immortalized cell line, the N9 microglia line, has also been used in cultures in an attempt to expedite bench work, while maintaining the crucial properties of *in vivo* microglia.

The N9 microglia is derived from mouse brain and shares many phenotypical characteristics with primary mouse microglia. This is exemplified by a number of studies including one by Hickman *et al.*[22]. Hickman’s group examined Aβ peptide clearance and receptor regulation of microglia by performing some experiments with the N9 cell line. They discovered that when the N9s were incubated with TNF- there were decreases in the expression of scavenger receptor A and CD36 and also reductions in Aβ uptake, supporting the results they obtained from primary mouse microglia. There is evidence that this cell line shares similarities with the primary microglia; however, they are genetically modified, which leads to increased proliferation and adherence.

The N9 microglia cells were developed by immortalizing primary microglia cells with the *v-myc* or *v-mil* oncogenes of the avian retrovirus MH2 [23]. The clones derived from this process were characterized as exhibiting nonspecific esterase activity and testing positive for microglia cell surface markers FcR, Mac-1 and F4/80. Furthermore, they are negative for GFAP, A2B5, and Gal-C. The microglia can also phagocytose opsonized sheep red blood cells (SRBCs) and readily produce cytokines. Upon stimulation with LPS, these cells produce IL-6, TNF- and IL-1 [23].

It has also been demonstrated that the N9 microglia line possesses purinergic receptors including the P2Y and P2Z subtypes, which are ATP sensitive. The P2Z receptor has been shown to be involved in IL-1β release in response to ATP stimulation [24]. In that study, the P2Z receptor was modulated by growing N9 cells in the presence of a high concentration of ATP, which selected for ATP-resistant clones. By comparing amounts of IL-1β released upon ATP stimulation from the cells with functional P2Z receptors and the ATP-resistant clones, it was concluded that the P2Z receptor was responsible for IL-1β release after ATP stimulation.

Establishing these purinergic receptor qualities in this cell line also indicates a similar Ca^2+^ signaling profile to that of primary microglia. Experiments such as these lend insight into the ability to passage immortalized microglia cells in the presence of trophic factors to select for a desired phenotype or modulate protein expression patterns.

These immortalized cell lines have been used to demonstrate the ability of microglia to change state and assume different roles in the AD brain, specifically in response to Aβ. The BV2 microglia have been shown to uptake Aβ by phagocytosis, a process that was increased dose dependently by specific neuropeptide incubation. Furthermore, the study found that the degradation of Aβ was unaffected. The intracellular enzyme level of neprilysin, which is thought to degrade Aβ, was lower after Aβ, suggesting that Aβ could negatively regulate the levels of neprilysin [25]. In agreement with research using primary microglia, the pro-inflammatory genes including *INOSCOX-2TNF-* and *IL-1β* were up-regulated [26]. Apart from gene levels, oligomeric Aβ has been shown to increase ROS and nitric oxide in the BV2 cells. The N9 microglia have also been shown to up-regulate the pro-inflammatory genes, similarly to the BV2, including *INOSCOX-2TNF-* and *IL-1β*[25]. Furthermore, Fu *et al.* reported a Mac-1/Complement component 3 mediated phagocytosis of fibrillar Aβ by N9 microglia [27].

This ability to employ the cell model to help answer specific inflammatory research questions can be valuable. Along the same line, it has often been argued that it is also important to use a cell line that is appropriate for the model system being used,(that is, N9 for the mouse model, BV2 for the rat model, and so on.). So, the human model, which is arguably one of the most important model systems, has its own cell line as well.

### Human immortalized microglia: HMO6

Human microglia systems are used in neuroscience research; however, it is more difficult to obtain these cells because they have to be derived from human embryos, which can be difficult to access due to the ethical and legal issues. Nagai’s group sought to circumvent this issue by creating an immortalized human microglia cell line, the HMO6 [28]. This cell line was established by using primary human embryonic microglia cultures. The cells were stimulated to proliferate by incubation with 8 μg/mL granulocyte-macrophage-colony-stimulating-factor in medium for seven to 12 days and then infected with the PASK 1.2 retroviral vector, which transcribed the *v-myc* oncogene. As a culture, the doubling time for these cells was quite fast, at 34.5 hours. The cells also adhered to glass or plastic and maintained the ability to phagocytose latex beads. Furthermore, HMO6 cells were positive for staining with *Ricinus communis* agglutinin (RCA) and CD11b (MAC-1) and express transcripts for purinergic receptors, confirming a microglia phenotype. Nagai’s group also performed RT-PCR analysis and showed gene expression of IL-1β, IL-6, IL-8, IL-10, IL-12, IL-15 and TNF-.

The characteristics of HMO6 cells make them appropriate for investigating pro-inflammatory processes in AD like pathology. Specifically, HMO6 cells have also been shown to express TNF- and IL-8. Following a 48-hour incubation with β peptide (25–35) or LPS. HMO6 cells increased gene expression and protein production of IL-8 and TNF- [28]. Furthermore, in a study in 2008, it was found that when HMO6 cells were treated with Aβ(1–42) or LPS the cells expressed high levels of the protein albumin, which has been implicated as a role player in the pathogenesis of AD [29].

These human-derived cell lines are rarely used based on the fact that it is difficult to obtain a primary culture to transfect. More research is conducted in non-human animals and, thus, murine microglia cultures are more commonly used.

### Spontaneously immortalized: EOC and HAPI

There are a few cell lines that offer highly proliferating microglia which are not genetically modified. Some common lines include the colony stimulating factor-1 dependant EOC cells [30] and also the Highly Aggressively Proliferating Immortalized (HAPI) cell line [31].

Similar to primary microglia, these cells possess the ability to secrete many cytokines and reactive species, including ·NO. The ·NO production of microglia is an important factor to consider in the pathogenesis of AD because it has been shown that Aβ(1–42) can cause significant production of NO_2_, a stable metabolite of ·NO [32]. Using the EOC-20 line, Hensley *et al.*[33] found that stimulation of microglia cells by 20 ng/mL recombinant TNF- causes a large NO_2_^-^ production, as measured by the Greiss reaction. It was also shown that the stimulated NO_2_^-^ production of EOC-20 cells can be inhibited by a CNS metabolite called lanthionine ketimine (LK) as well as its synthetic ether derivative, LKE [33]. The modulation of ·NO production using these microglia can have important implications for future research in neurodegenerative disorders where ·NO is known to be associated with pathological conditions.

**Figure 1:**
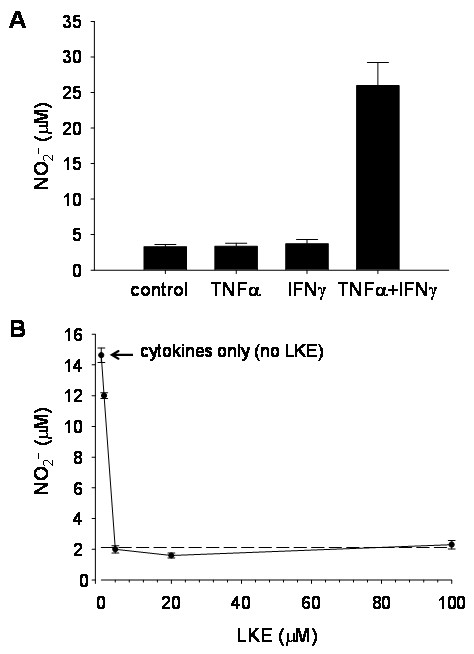
**·NO production in EOC-20 cells.A**, EOC-20 microglia stimulated only with TNF + IFNγ in combination. Each error bar represents mean ± SEM from six wells of a typical experiment. **B**, LKE inhibits cytokine-stimulated ·NO production in EOC-20 microglia challenged with 40 ηg/mL TNF + 40 ng/mL IFNγ. Each point represents mean ± SEM from four wells of a typical experiment. SEM, standard error of the mean.

Mouse-derived EOC cells are available from American Type Culture Collection (ATTC) in subtypes EOC-2, EOC-13.31, and EOC-20. The numbers following EOC are designated by the colonies they were originally derived from within the first culture by Walker *et al.*[30]. The major difference is that the EOC-2, unlike EOC-13.31 and EOC-20, does not express major histocompatibility complex class II (MHCII). Furthermore, the MHCII is up-regulated by recombinant IFN-γ in EOC-20 cells but not in EOC-13.31. These differences are often considered when using cell lines to investigate differential activation by cytokines in neurodegenerative research. Specifically, Hensley’s research group noted a TNF--induced NO_2_^-^ production in EOC-20 cells, which was potentiated by co-incubation with TNF- plus IFN-γ or IL-6 [34]. Furthermore, in recent experiments the Hensley laboratory has found that the ·NO production and up-regulation of iNOS is dependent on both TNF- and IFN-γ in combination (Figure 1). This change in cytokine activation may be attributed to cell strain evolution during repeated passaging and freeze-thaw storage procedures or unspecified laboratory level differences. This situation also highlights the ability of immortalized cells to change phenotypically over time. These cells are easily cultured; however, they do require colony-stimulating factor-1 (CSF-1). CSF-1 can be obtained from a separate cell line, the bone marrow derived LADMAC (ATCC® CRL-2420) which produce this factor. So in order to culture EOC cells, LADMAC cells are required to complete the EOC conditioned medium. The similarly spontaneously immortalized cell line, the HAPI microglia, is derived from rat and is not dependent on a specific growth factor to maintain immortalization.

The HAPI cell line was first recognized to be spontaneously immortalized by Cheepsunthorn *et al.* and was observed to be highly proliferating within an enriched primary microglia culture [31]. One day after primary microglia purification by the ‘shake off’ method described by Giulian and Baker [8], cells were harvested and cultured in 10% fetal bovine serum. These cells possess the ability to phagocytose, as well as test positive for OX42, which recognizes complement 3 receptors, indicating a phenotype typical of microglia. Furthermore, the cells were negative for A2B5 and GFAP.

To further characterize this cell line, Cheepsunthorn *et al.*[31] used RT-PCR analysis to discover that treatment with LPS up regulates proinflammatory cytokines such as TNF- and iNOS mRNA in HAPI cells. In addition to this, it was also found that in response to LPS, these cells produce significant quantities of ·NO and TNF- in the spent culture medium. Furthermore, methamphetamine stimulation produces several cytotoxic factors including IL-1β, IL-6, TNF-, and ROS/RNS [35]. This methamphetamine activation of microglia which results in cytokine and ·NO release is reduced by melatonin pretreatment which also increased cell viability in cultures [35].

Similar to inflammogen stimulants such as LPS and methamphetamine, Aβ also has the ability to activate EOC microglia. When EOC cells were exposed to Aβ(24–35) and incubated with carboxy-H_2_DCFA, Manzano-Leon *et al.* found an increase in fluorescence after 24 hours, indicating an increase in production of ROS [36]. This rise in ROS was suggested to alter the protein β-adaptin, a molecule needed to organize the endocytic machinery in microglia, and contribute to the lack of Aβ clearance in AD. In HAPI cells, Aβ(1–42) induces serine racemase, the enzyme that converts L-serine to D-serine. D-Serine then acts on neuronal NMDA receptors as a signaling molecule [37]. Although there have been some studies done using EOC and HAPI microglia to examine Aβ effects, the amount of published research is relatively small in comparison with primary cultures or other immortalized cell lines.

### Future directions of microglia culture experimentation

In any reductionist approach, experiments that lack the entire brain milieu will leave gaps in translatability to *in vivo* situations and thereby be subject to scrutiny. However, by minimizing surrounding variables, it allows researchers to study functional aspects of microglia unencumbered by those surrounding factors that may make it difficult to tease apart microglia function from other cells in proximity. A variety of microglial cell lines are now used routinely for research purposes, in addition to primary microglia. Table 2 summarizes the principal characteristics of the common microglial culture systems described in this review, with special attention to their sensitivity towards Alzheimer’s disease-associated amyloid beta-peptides.

**Table 2:** Microglia properties by cell line

	Primary Microglia	BV2	N9	HMO6	EOC	HAPI
MAC-1	+	+	+	+	+	+
LPS stimulation	+	+	+	+	+	+
lL-1β release	+	-	+	-	N/A	+
TNF- release (Following LPS)	+	+	+	+	N/A	+
Phagocytosis	+	+	+	+	+	+
Peroxidase	-	-	-	-	-	-
Non-specific esterase	+	+	+	+	+	+
GFAP	-	-	-	-	-	-
GC	-	-	-	-	-	-
·NO production	+	+	+	-	+	+
Aβ induced lL-β	+	+	+	-	N/A	N/A
Aβ induced TNF-	+	+	+	+	N/A	N/A
Aβ phagocytosis	+	+	+	N/A	N/A	N/A
Number of articles in Pub Med^a^	302	142	91	8	18	19

The table represents properties of individual cell lines as reported by citations in the PubMed Database (http://www.ncbi.nlm.nih.gov/). (+) Positive for trait, (−) negative for trait. ^a^Specific PubMed search term ‘primary microglial cell culture’ (from year 2000 to present; includes both studies of endogenous microglia as well as primary cultures of microglial isolates). Aβ, amyloid β; GC, galactocerebroside; GFAP, glial fibrillary acidic protein; LPS, lipopolysaccharide.

One way to add another dynamic to microglia culture systems, which can help assess microglia interaction with other cell types, is co-cultures. Several studies have been published in which microglia are cultured with neurons and/or astrocytes to study the interaction of these cells. This allows for the *in vitro* environment to be controlled as far as temperature, medium content and other factors. Laboratories studying neurodegenerative diseases with neuron cultures can benefit from these co-cultures as well, because microglia have been shown to have many interactions with the micro-environment.

It is known that in AD high concentrations of Aβ(1–40) or Aβ(1–42) do not cause neuronal damage if microglia are not present [1]. So, by utilizing co-culture methods, Li *et al.* activated microglia with Aβ precursor protein or LPS and then placed the microglia with neocortical neurons [38]. They found that in conditions where activated microglia were present, there was a significant increase in phosphorylation of neuronal tau and a decline in synaptophysin levels, similar to tangles found in the AD. Furthermore, in regards to microglia activation, co-cultures of microglia with motor neurons that were treated with IL-4, showed a suppression of the M1 microglial activation, which reduced the release of ROS and improved motor neuron survival [39]. These situations highlight some of the ways in which microglia co-cultures can be very useful in neurodegeneration research.

Microglia cell lines certainly do not come without faults. One problem may be with selection of certain cell characteristics that differ between laboratories due to medium or culturing conditions [19]. This can lead to similar treatment conditions yielding far different cell responses. One instance of this was the study of TNF- expression and release in HAPI microglia by Cheepsunthorn *et al.*, [31] and Horvath *et al.*[18]. While characterizing these cells, both groups stimulated HAPI microglia with LPS, and Cheepsunthorn *et al.*[31] found an increase in TNF- expression and release. Similarly, Horvath *et al.*[18] found increased expression at 24 and 48 hours following LPS treatment; however, there was no release of TNF- into the medium at either time point. Horvath *et al.*[18] reasoned that the discrepancy may have occurred due to genetic drift of the cell line or possibly technical differences in the experimental preparation. This is an example of how cell lines may react differently in different hands due to many different factors. It also sheds light on the immortalized cell lines ability to genetically drift or select for certain phenotypes, as they are passaged many times over. To control for this, culture techniques and methods must become more cohesive and standardized within the research community and supply companies.

The biological functioning of microglia in many brain pathologies, especially AD, has led to the development of several cell lines. While primary microglia cultures tend to be the closest to *in vivo* microglia, they are more tedious and technically time consuming to prepare. Also, the populations may not be completely homogeneous for microglia because astrocytes or oligodendrocytes may also remain in the culture. Conversely, immortalized cell lines tend to be a high throughput model for experimentation and are completely homogeneous throughout the culture. This comes at the cost of having an inflammatory response that is not identical to primary cultures. Also, the immortalized cells are subject to genetic drift and morphology changes. Taken together, these microglia cell lines share common strengths, with the goal of all cell lines being to provide the best tool for the study of microglia-related phenomena, and to help understand brain diseases.

## Abbreviations

Aβ, Amyloid β-peptide; AD, Alzheimer’s disease; ALS, Amyotrophic lateral sclerosis; ATCC, American Type Culture Collection; ATP, Adenosine triphosphate; BV2, BV2 retroviral-immortalized microglia; CNS, Central nervous system; COX-I/II, Cyclooxygenase I/II; CSF-1, Colony stimulating factor-1; EOC, EOC spontaneously immortalized microglial cell lines; GC, Galactocerebroside; GFAP, Glial fibrillary acidic protein; HAPI, Highly aggressively proliferating immortalized microglial cell line; H2DCF, Reduced dichlorofluorescein diacetate; HMO6, Human immortalized microglial cell line; IL-1β, Interleukin-1β; IL-6/8/10/12/15, Interleukin-6/8/10/12/15; IFNγ, Interferon-gamma; LADMAC, LADMAC colony stimulating factor-producing cell line; LK, Lanthionine ketimine; LKE, Lanthionine ketimine-5-ethyl ester; iNOS, Inducible nitric oxide synthase (NOS-II); LPS, Lipopolysaccharide; MAC-1, Macrophage antigen-2 (CD11b); MHC, Major histocompatibility class; M1, Classically-activated microglial phenotype; M2, Alternatively-activated microglial phenotype; N9, N9 retroviral-immortalized microglia; ·NO, Nitric oxide; NOS, Constitutive nitric oxide synthase (NOS-1); NOX, NADPH oxidase; PD, Parkinson’s disease; P2Y/P2Z, Purinergic receptor subtypes; RCA, Ricinus communis agglutinin; RNS, Reactive nitrogen species; ROS, Reactive oxygen species; SRBC, Sheep red blood cells; RT-PCR, Reverse transcriptase-polymerase chain reaction; TNF-, Tumor necrosis factor-alpha.

## Competing interest

The authors declare that there are no competing interests.

## Authors' contributions

BS and JP cultured microglia, performed experiments discussed in the manuscript, and drafted the manuscript. KH supervised laboratory experiments, instructed manuscript format and edited the final manuscript. All authors read and approved the final manuscript.
